# A highly sensitive colorimetric approach based on tris (bipyridine) Ruthenium (II/III) mediator for the enzymatic detection of phenylalanine

**DOI:** 10.3389/fchem.2023.1164014

**Published:** 2023-04-10

**Authors:** Maria Anna Messina, Ludovica Maugeri, Giuseppe Forte, Martino Ruggieri, Salvatore Petralia

**Affiliations:** ^1^ Expanded Newborn Screening Laboratory, A.O.U Policlinico “G. Rodolico—San Marco”, Catania, Italy; ^2^ Department of Drug and Health Sciences, University of Catania, Catania, Italy; ^3^ Department of Clinical and Experimental Medicine, University of Catania, Catania, Italy

**Keywords:** phenylalanine, enzymatic-sensing, phenylketonuria, tris (bipyridine) Ruthenium (II/III), colorimetric-assay

## Abstract

The accurate monitoring of phenylalanine concentration plays a prominent role in the treatment of phenylketonuria (PKU). In this study, we present an enzymatic assay based on Phenylalanine Dehydrogenase/NAD^+^ and tris (bipyridine) Ruthenium (II/III) as a colorimetric mediator for the detection of Phenylalanine concentration. The amount of amino acid was quantitatively recognized by optical absorption measurements at 452 nm through the conversion of Ru (byp)_3_^3+^ to Ru (byp)_3_^2+^, which is induced by the neoformed NADH. A detection limit of 0.33 µM, a limit of quantification of 1.01 µM, and a sensitivity of 36.6 a.u nM^−1^ were obtained. The proposed method was successfully tested using biological specimens from patients affected by hyperphenylalaninemia. The proposed enzymatic assay showed a high selectivity, making it a promising alternative for the development of versatile assays for the detection of phenylalanine in diluted serums.

## 1 Introduction

Hyperphenylalaninemia (HPA), including phenylketonuria (PKU), is a class of diseases caused by the impairment of the enzyme Phenylalanine hydroxylase (PAH), both for mutations in the corresponding *PAH* gene encoding the enzyme and for deficiency of the BH_4_ cofactor (Shintaku, 2002). The enzyme deficiency results in the accumulation of phenylalanine (Phe) in the blood, causing brain damage, intellectual disability, and neurological problems. There is no cure for HPA, but early treatment can prevent intellectual disabilities and other health issues (PubMed Health, 2011). Patients with PKU require a severe diet that limits the intake of Phe (Poustie and Wildgoose, 2010). To date, Phe is the main biomarker used to monitor the progress of the PKU disease. Therefore, given the importance of frequently monitoring Phe levels’ intake with meals, a straightforward growth in the development of new analytic assays (Matulis and Naglis Malys, 2023), sensors (Dinu et al., 2022), and wearable devices (Parrilla et al., 2022) for Phe recognition was observed. The integration of the assay into a miniaturized device is a critical key for the setup of effective and multiplexing point-of-care platforms (Ventimiglia and Petralia, 2013). In this context, a great effort has been focused on the development of innovative nanostructured materials for effective assay miniaturization. Recently, our team developed novel photothermal nanomaterials based on β-CD/Co_3_O_4_ for the photothermal activation of Phe conversion into phenylpyruvate mediated by the Phenylalanine Dehydrogenase (PDH) NAD-dependent enzyme (Maugeri et al., 2022). Similarly, non-enzymatic and mediator-free approaches for the detection of Phe have been developed. They include methods based on Surface Plasmon Resonance imprinted polymers (Cimen et al., 2021) and on the recognition of Phe by interacting with specific DNA-aptamers sequences (Cheung et al., 2019). Despite great efforts in the development of enzyme-mimics nanosystems, the excellent specificity of the enzymatic reactions together with the good performance of the redox/colorimetric mediators makes enzymatic-based-assay the most used approach for the detection of Phe. Phenylalanine ammonia lyase (PAL) was the first investigated enzyme. It converts Phe to *trans*-cinnamic acid, producing a quantitative amount of ammonia, which can be detected by direct optical measurements (Sari et al., 2022) through an easy color change of the pH indicator (Villalonga et al., 2008) or by capacitive measurements using zirconia oxide nanostructures (Ando et al., 2022). The PDH is the most investigated NAD-dependent enzyme used for the development of enzymatic-analytical assays for Phe recognition. It converts, specifically, Phe, in the presence of NAD^+^, to Phenylpyruvate and NADH (nicotinamide adenine dinucleotide hydrogen). The direct optical spectroscopic measurement of neo-formed NADH at 340 nm is limited by its low absorption coefficient value. To overcome this drawback, a great effort has been made to develop novel transduction processes based on specific reactions with mediators, producing optical or electrical signals. Various electrochemical and colorimetric mediators, including the cationic dye MTT^¥^, which is converted, in the presence of NADH and diaphorase enzyme, to the colored formazan have been reported in previous works of literature (Jafari et al., 2021). Similarly, methoxy phenazine-methosulfate and nitroblue tetrazolium are converted by NADH to colored formazan derivatives (Robinson et al., 2016). Although good results in terms of Lod and sensitivity were obtained, the not-quantitative colorimetric reaction requires additional enzymatic steps, making the protocol more cumbersome. Recently, our team has developed an enzymatic assay based on the PAL enzyme for the measurement of Phe levels. The Phe amount was easily recognized through pH-indicator color changes induced by the ammonia formation. The system permits the monitoring of Phe in a dynamic range concentration of 10–3,000 μM with a limit of detection of approximately 19 µM. The assay was successfully integrated into a miniaturized paper-based lab-on-chip device (Messina et al., 2017). In this study, we reported an innovative colorimetric-enzymatic assay based on tris (bipyridine) Ruthenium (II/III) complex (Ru (byp)_3_^2+/3+^) as a colorimetric-redox mediator through the PDH/NAD^+^ enzyme. The amount of NADH formed is revealed by the reaction with the colorimetric Ru (byp)_3_^3+^ mediator through the formation of an intense absorption band centered at 452 nm. The method was successfully tested using human blood from patients affected with HPA.

## 2 Experimental sections

### 2.1 Materials and methods

All reagents including L-Phenylalanine (Phe) 98% (M.W. = 165.19 gr mol^−1^), *Phenylalanine Dehydrogenase* (PDH) from Sporosarcina sp (6.0 U/mg), NAD+ (M.W. = 663.43 gr mol^−1^), and Tris (2,2′-bipyridyl) dichlororuthenium (II) were purchased from Sigma-Aldrich in their highest purity and used without further purification. MilliQ-water was used in all experiments. The L-amino acid spectrophotometric commercial quantitation kit (cod. ACMAK002KT) was used. UV-Vis-NIR spectroscopic analyses in transmission mode were carried out on the Perkin-Elmer 365 using a standard quartz cuvette with an optical length of 1 cm. The plasma separation process was performed on the Vivid Plasma Separation Membranes (PALL, thickness, 300 ± 20 μm, and Grade GR). They were handled as received, and no pre-treatment was performed.

### 2.2 Colorimetric mediator solution preparation

The mediator Ru (byp)_3_^3+^ solution was prepared by oxidation reaction of the commercial Ru (byp)_3_Cl_2_ product with Cerium (IV) ammonium nitrate (CAN), which is a strong one-electron oxidant (E_1/2_ = 1.6 V vs*.* NHE) (Pérez-Ruiz, et al., 2003). It follows the reaction. Ru(byp)_3_^2+^ + Ce(NO3)_6_^2−^ → Ru(byp)_3_^3+^ + Ce(NO3)_6_^3−^

In detail, to a volume of 2 mL of Tris (2,2′-bipyridyl) dichlororuthenium (II) (Ru (byp)_3_Cl_2_) (7 mg/mL) was added a portion of 2 mL of cerium (IV) ammonium nitrate (CAN) with continuous stirring at room temperature. After 30 min, the solution was filtered (0.2 µm filter pore size) and used. The CAN stock solution was prepared by dissolving an amount of 25 mg of product into 4 mL of hydrochloride acid (10 mM).

### 2.3 Enzymatic-assay procedure

To a volume of 1.0 mL of phosphate buffer saline (PBS) at a pH value of 8.3 were added 150 µL NAD+ (75 mM) and 0.2 U of the PDH enzyme. Various amounts of Phe were added to obtain a final concentration ranging from 1 μM to 12.7 µM. The mixture was incubated at 37°C for 30 min then Ru (byp)_3_^(3+)^ colorimetric mediator solution (30 µM) was added with continuous stirring, and the UV-Vis optical spectra were recorded after 10 min of incubation at 24°C. The same procedure was used for the testing of filtered human plasma samples. Each analysis was replicated three times.

### 2.4 Plasma separation procedure

The plasma separation was conducted using the polysulfone membrane Grade GR. An amount of 20 µl of fresh human blood was deposed on the membrane disk (diameter of approximately 8 mm), and after 3 min of filtration, a volume of approximately 7–10 µl of plasma sample was collected and tested. Each analysis was replicated three times.

## 3 Results and discussion

### 3.1 Enzymatic-assay mechanism

**SCHEME 1:**
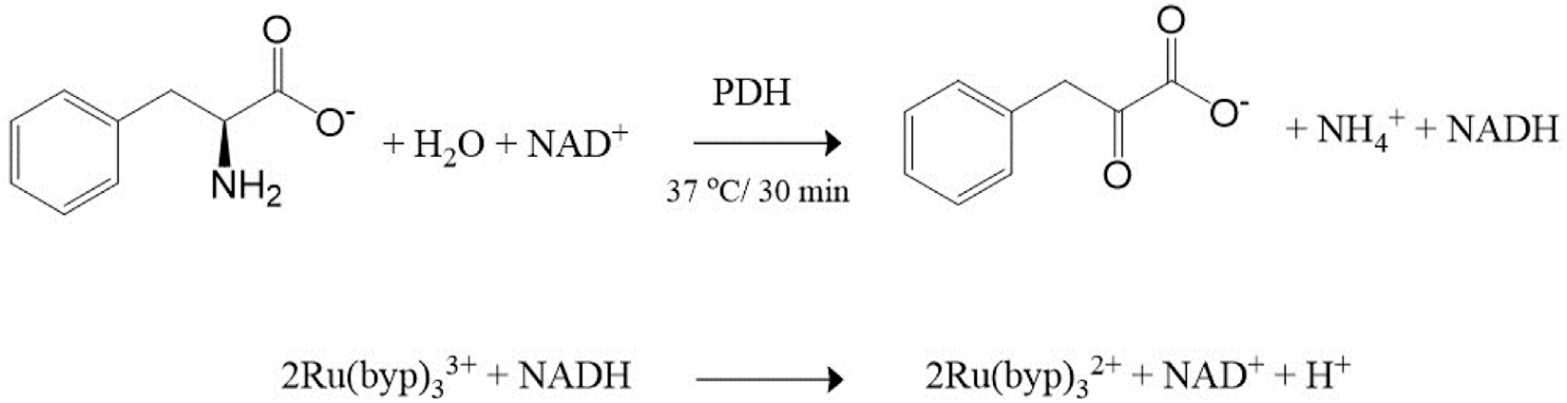
Phe sensing mechanism: (1) Enzymatic reaction catalyzed by PDH/NAD^+^ and (2) colorimetric transduction process based on Ru (byp)_3_^2+/3+^ mediator.

**FIGURE 1:**
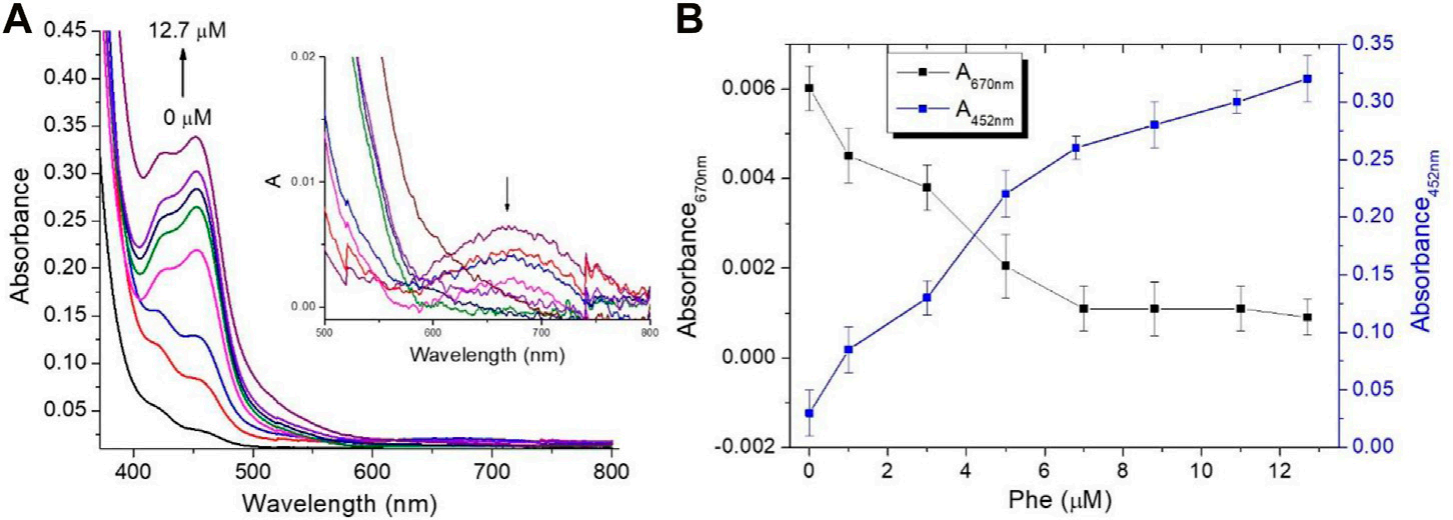
Spectroscopic measurements for the mixture assay at different Phe concentrations (0, 1.0, 3.0, 5.0, 6.8, 8.8, 10.9, and 12.7 µM) **(A)** Optical absorption changes in PBS, and the presence of a Ru-complex mediator concentration of 30 µM insets the optical absorption spectra change in the region from 500 to 800 nm and **(B)** Absorbance values at 452 nm and 670 nm at different Phe amounts.

Based on the experimental observations and literature data, the working mechanism for the sensing of Phe using Ru (III/II) complexes mediator is proposed in Scheme 1. Firstly, the enzymatic reaction converts Phe into phenylpyruvate through PDH/NAD^+^ with NADH formation (Reaction 1). The neoformed NADH was spectroscopically confirmed through the appearance of the absorption band centered at 340 nm (Supplementary Figure S1). The second step involves one-electron transfer from NADH to the Ru (byp)_3_^3+^ in an outer sphere manner to yield the Ru (byp)_3_^2+^ and the NADH^•+^ radical. The reduction of another molecule of the Ru(III) complex by NADH•^+^ radical occurred in a subsequent rapid and kinetically inconsequential step (Reaction 2) (Chrzanowska et al., 2022). The conversion of Ru (byp)_3_^3+^ to Ru (byp)_3_^2+^ is revealed by the formation of the metal-ligand charge transfer (MLCT) absorption band centered at approximately 452 nm (Figure 1A).

The proposed mechanism in the reduction of a similar Ru^3+^complex system induced by NADH was supported by recent literature data (Reyes and Tanaka, 2017). Moreover, to confirm the role of NAD^+^ on the mechanism, additional experiments were carried out in the absence of NAD^+^ species. In this case, no Ru^III^/Ru^II^ complexes conversion was observed; whereas, in the presence of NAD^+^ (10 µM), the reduction in conversion arose as confirmed by the formation of the optical absorption band centered at 452 nm (Supplementary Figure S2). The optical properties, in terms of optical absorption and emission spectra for the Ru (byp)_3_^3+/2+^ mediator, are reported in Supplementary Figure S3.

To assess the potential of the proposed colorimetric mediator, various Phe amounts (0, 1.0, 3.0, 5.0, 6.8, 8.8, 10.9, and 12.7 µM) were investigated. Figure 1A illustrates the optical absorption changes of the assay at different Phe amounts in the presence of Ru (byp)_3_^3+^ mediator concentration of approximately 30 µM. It is evident that there is a conversion of Ru (byp)_3_^3+^ to Ru (byp)_3_^2+^ as Phe amounts increase, as confirmed by the formation of the absorption band centered at 452 nm and by the disappearance of the absorption band centered at 670 nm for the Ru (byp)_3_^3+^ (Figure 1A-inset). Figure 1B illustrates the change in the absorbance values recorded at 452 nm and 670 nm at different Phe levels. 

### 3.2 Analytical parameters assessment

**FIGURE 2:**
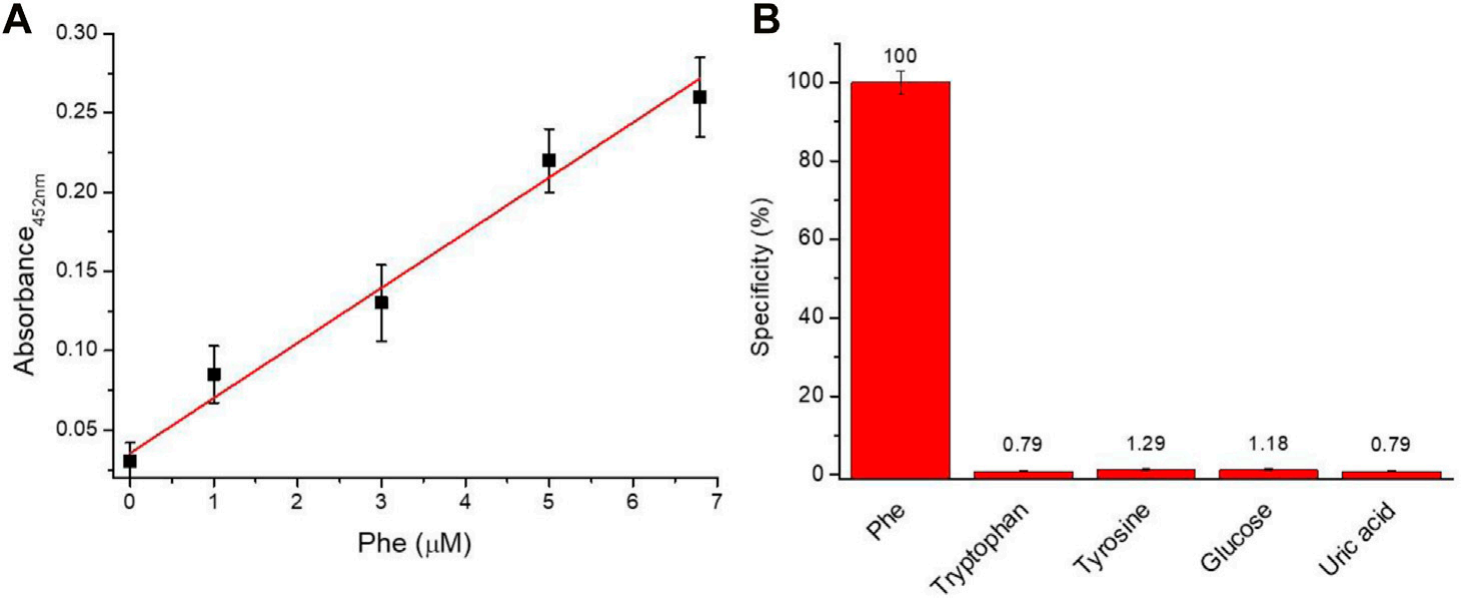
Analytical performance **(A)** Linearity response of the assay (*Y* = 0.0356 + 0.04821X; *R* ^2^ = 0.9958) and **(B)** Selectivity (%) of Phe assay versus different biomolecules.

In order to evaluate the analytical performance of the proposed assay, linear response range, sensitivity, the limit of detection (LoD), the limit of quantification (LoQ), and selectivity were investigated. Figure 2A illustrated the linear relationship between the absorbance value at 452 nm and the amount of Phe. A dynamic linear range of 0–7 µM was observed with a sensitivity of approximately 36.6 a.u. nM^−1^. Moreover, an LoD of 0.33 µM and LoQ of 1.01 µM were obtained using the following formulas: LoD = 3σ/slope and LoQ = 10σ/slope (where the background standard deviation *σ* is 0.00372). The capability of the assay to selectively discriminate between Phe versus other molecules was investigated. In detail, the assay was tested in the presence of tryptophan, tyrosine, glucose, and uric acid at a concentration of 12.7 µM. The data reported in Figure 2B confirm the high specificity (∼98.5%) of the proposed assay for the recognition of Phe.

### 3.3 Phe detection in human blood

To assess the capability of the proposed assay to measure Phe concentrations in human specimens, an aliquot of fresh whole blood (20 µl) was deposed on the membrane filters (diameter of approximately 8 mm) (Supplementary Figure S4). After 3 min, a volume of approximately 7–10 µl of plasma samples was collected and the amount of Phe was measured in a final volume of 200 µl as reported in the experimental section. Phe concentration value of approximately 3.6 ± 0.2 µM was obtained by calibration line interpolation (Figure 2A). Phe concentration, corrected by the dilution and filtration factors, corresponds to a final Phe concentration of 51.6 ± 2.0 µM on the whole blood sample. The proposed method was successful, compared with the commercial quantification amino acid kit which discloses a final Phe concentration of 53.5 ± 3.0 µM. The analysis was replicated three times.

### 3.4 Comparison of Phe detection methods

Various enzymatic and non-enzymatic approaches for the detection of Phe have been recently developed and reported in previous works of literature. These are mainly based on colorimetric and electric detections and some of these were successfully integrated into miniaturized devices for the development of portable systems. Table 1 reports a comparison between the different approaches recently reported in previous works of literature for the recognition of Phe. Non-enzymatic approaches based on the electric recognition of Phe by DNA-aptamers have been independently reported by Cheung et al. (2019) and Hasanzadeh et al. (2018). Both works report excellent sensitivity and good specificity, but the assays’ preparation requires laborious procedures and is hard to integrate into a miniaturized and disposable device. Among the enzymatic approaches, PAL and PDH are the most investigated enzymes. Recently, a colorimetric paper-based biosensor based on the PDH enzyme was proposed by Jafari et al. (2021) for the highly sensitive and selective determination of Phe, using MTT^¥^ and NBT^¥¥^ as mediators, reaching an LoD of 66.5 µM and 34.5 µM, respectively. In addition, different patents for Phe recognition were recently granted. Nakamura and co-inventors report a PDH/NAD enzymatic approach using 1-Methoxy-PMS as an electrochemical mediator (Nakamura et al., 2002). This method was successfully integrated into a disposable device reaching an LoD of 5.0 µM. An innovative enzymatic-sensing method using a carbon-based colorimetric mediator for Phe recognition in the dry film was invented by DeSilva et al. (2006). Despite the good sensitivity and the easy testing procedure, the dynamic range of 1–15 mM obtained with real samples would limit its diffusion. Recently, an alternative approach based on the aptamer/organometallic receptor was reported by Yang and colleagues for the electrochemical determination of phenylalanine. A linear range of 10–260 µM and an LoD of 1.03 µM were declared by the authors Yang et al. (2023).

**TABLE 1:** A comparison of different methods for Phe detection.

Detection method	LoD	References
Enzymatic (PAL)/colorimetric pH indicator	∼19 µM	Messina et al. (2017)
Enzymatic (PDH/NAD^+^)/MTT-AuNPs colorimetric mediator	34.5 µM	Jafari et al. (2021)
Enzymatic (PDH/NAD^+^)/NBT colorimetric mediator	66.5 µM	Jafari et al. (2021)
Enzymatic (PDH/NAD^+^)/Ru^II^ colorimetric mediator	330 nM	This work
DNA aptamers coupled with Nansized-In_2_O_3_/electric detection	Range ∼ fM	(Cheung et al., 2019)
DNA aptamer immobilized gold nanostructure/electrochemical detection	0.23 µM	Hasanzadeh et al. (2018)
Enzymatic PDH/NAD/1-Methoxy-PMS colorimetric mediator	5.0 µM	Nakamura et al. (2002)
Enzymatic PDH/NAD/1-Methoxy-PMS colorimetric mediator in the dry film	50 µM	DeSilva et al. (2006)
Aptamer/organometallic receptor-based methodology for label-free and amplified electrochemical determination of phenylalanine	1.03 µM	Yang et al. (2023)

Compared with previously published approaches, our proposed method shows excellent sensitivity and good LoD together with high specificity (> 98.5%). These parameters are crucial to allow effective integration of the assay on the microfluidics system.

## 4 Conclusion

In summary, a highly sensitive enzymatic approach based on PDH/NAD^+^ and Ru-complexes colorimetric mediator was demonstrated for the sensitive and convenient quantification of Phe. This approach can measure Phe concentrations in diluted filtered plasma by optical absorption measurements at 452 nm through the enzymatic conversion of Phe in phenylpyruvate. The neoformed NADH induces the quantitative conversion of Ru (byp)_3_^3+^ in Ru (byp)_3_^2+^ species with the formation of the MLCT absorption band centered at 452 nm. The determination of Phe with a limit of detection of 0.33 µM, a limit of quantification of 1.01 µM, and a sensitivity of 36.6 a.u nM^−1^ was demonstrated. Compared with traditional methods, the employment of colorimetric mediators improves detection sensitivity and selectivity. Considering these advantages, this approach should be integrated into a miniaturized biosensor for the self-testing of Phe concentrations, paving the way for the future development of PoC platforms. The system should be an excellent improvement to the quality of life of patients with HPA through the monitoring of diet therapy for HPA disorders in real-time.

### 4.1 Notes

^¥^ MTT= (3-(4,5-dimethylthiazol-2-yl)-2,5-diPhenyltetrazolium bromide).

^¥¥^ NBT = nitro blue tetrazolium.

## Data Availability

The original contributions presented in the study are included in the article/Supplementary Material. Further inquiries can be directed to the corresponding author.
