# Supplementary material for: A highly sensitive colorimetric approach based on tris (bipyridine) Ruthenium (II/III) mediator for the enzymatic detection of phenylalanine
Source: Front Chem. 2023 Apr 10;11:1164014. doi: 10.3389/fchem.2023.1164014 (PMC10123266; doi:10.3389/fchem.2023.1164014)
Supplement: Supplementary file 1 [file DataSheet1.docx]

Supplementary Material for

A highly sensitive colorimetric approach based on tris(bipyridine)Ruthenium (II/III) mediator for the enzymatic detection of Phenylalanine

Maria Anna Messina,^1^ Ludovica Maugeri^2^, Giuseppe Forte^2^, Martino Ruggieri^1^, and Salvatore Petralia* ^2^

^1^Expanded Newborn Screening laboratory, A.O.U Policlinico “G. Rodolico San Marco”, Via Santa Sofia 78, 95125 Catania, Italy.

^2^ Department of Drug and Health Sciences, University of Catania, Viale Andrea Doria 6, 95125 Catania, Italy

^3^ Departmemt of Clinical and Experimental Medicine, University of Catania

*** Correspondence:** Salvatore Petralia ([salvatore.petralia@unict.it](mailto:salvatore.petralia@unict.it) ).

**Figure SI1** Optical absorption spectra for the reaction 1 at various amount of Phe. The formation of absorption band at 340 nm confirms the formation of NADH

**Figure SI2** Optical absorption spectra for the Phe enzymatic-assay detection via Ru(II)/Ru(III) mediator (30 µM) without (I) and with (II) cofactor NAD^+^ (10 µM).

**Figure SI3** Optical absorption spectrum for Ru(byp)_3_^(II)^ (5x10^-5^M) line 1, fluorescence emission spectrum for Ru(byp)_3_^(II)^

(5x10^-5^M) line 2 and optical absorption spectrum for Ru(byp)_3_^(III)^ (6x10^-4^ M) line 3.


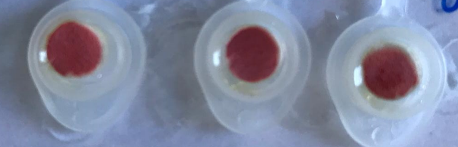


**Figure SI4** Plasma separation process on Vivid Plasma Separation membrane (Grade GR).

**Figure SI5** Optical absorption spectra for the Phe enzymatic-assay detection of human specimen
